# Supplementary figures and images for: How well do rudimentary plasticity rules predict adult visual object learning?
Source: PLoS Comput Biol. 2023 Dec 11;19(12):e1011713. doi: 10.1371/journal.pcbi.1011713 (PMC10754461; doi:10.1371/journal.pcbi.1011713)

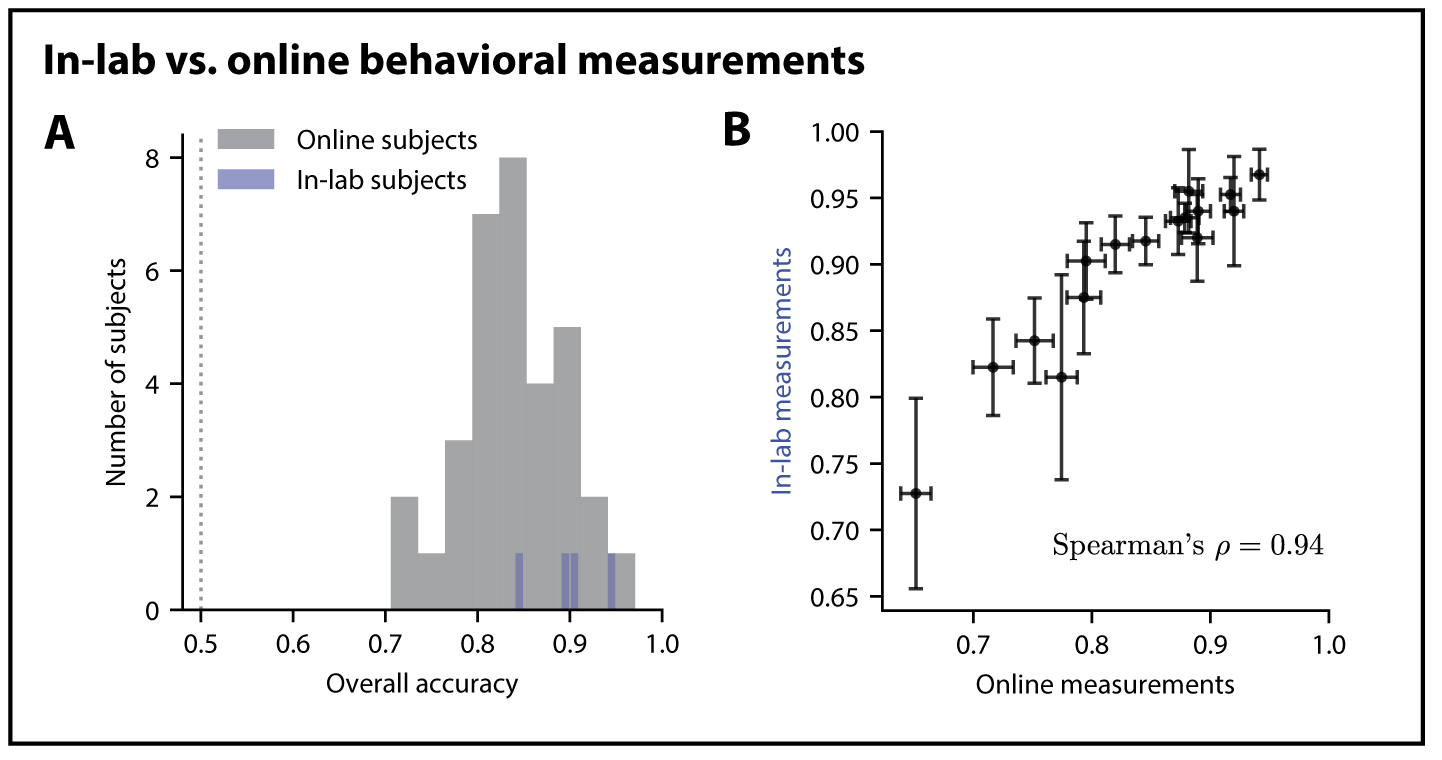

Supplement: S1 Fig — We took measurements for n = 16 randomly selected subtasks from Experiment 1 in a group of in-lab human subjects (n = 4) that used a chinrest and calibrated monitor setup. In A, we show that the overall accuracy of these in-lab subjects fell within the empirical support of the subject distribution from our online experiments. In B, we show that patterns of average accuracy (over subtasks) were tightly correlated between the in-lab and online populations (Spearman’s ρ = 0.94; see Section 1.1 in S1 Appendix). Errorbars are SEM (simple bootstrap over subjects). (TIF) [file pcbi.1011713.s001.tif]

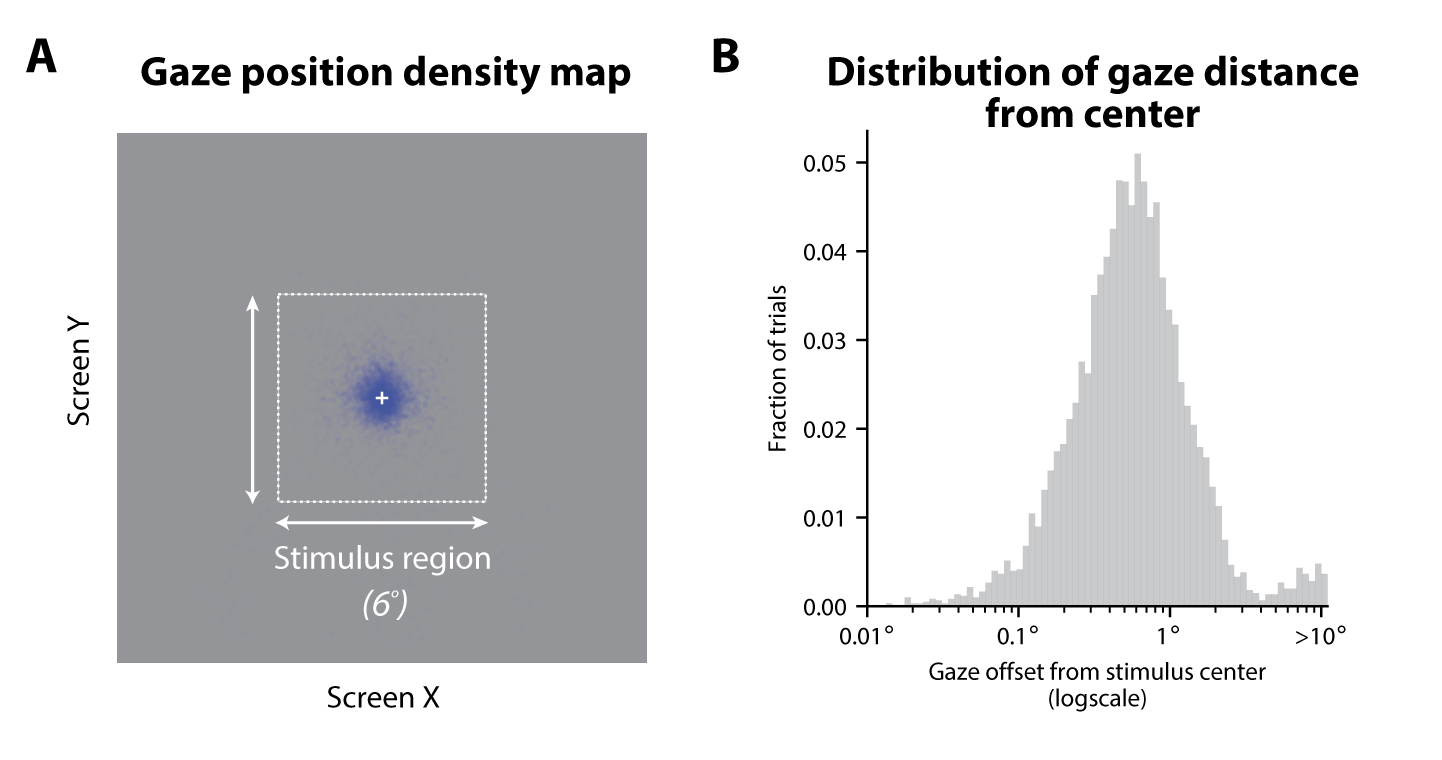

Supplement: S2 Fig — We passively recorded eye movements from our in-lab subjects using an Eyelink 1000 Plus (monocular; desktop mounted) as they performed the task. In A is the overall distribution of the subjects’ gaze position (shown in blue) at the time of onset of stimulus presentation (i.e. the distribution over subjects and trials). In B is the distribution of gaze distance from the stimulus center (logscale) over all subjects and trials; the median distance from the center of the stimulus was 0.57° ± 0.13° (mean ± standard deviation over subjects). We found that on ≈ 95% of trials, the subject’s gaze was located in the test image region when it appeared on the screen. (TIF) [file pcbi.1011713.s002.tif]

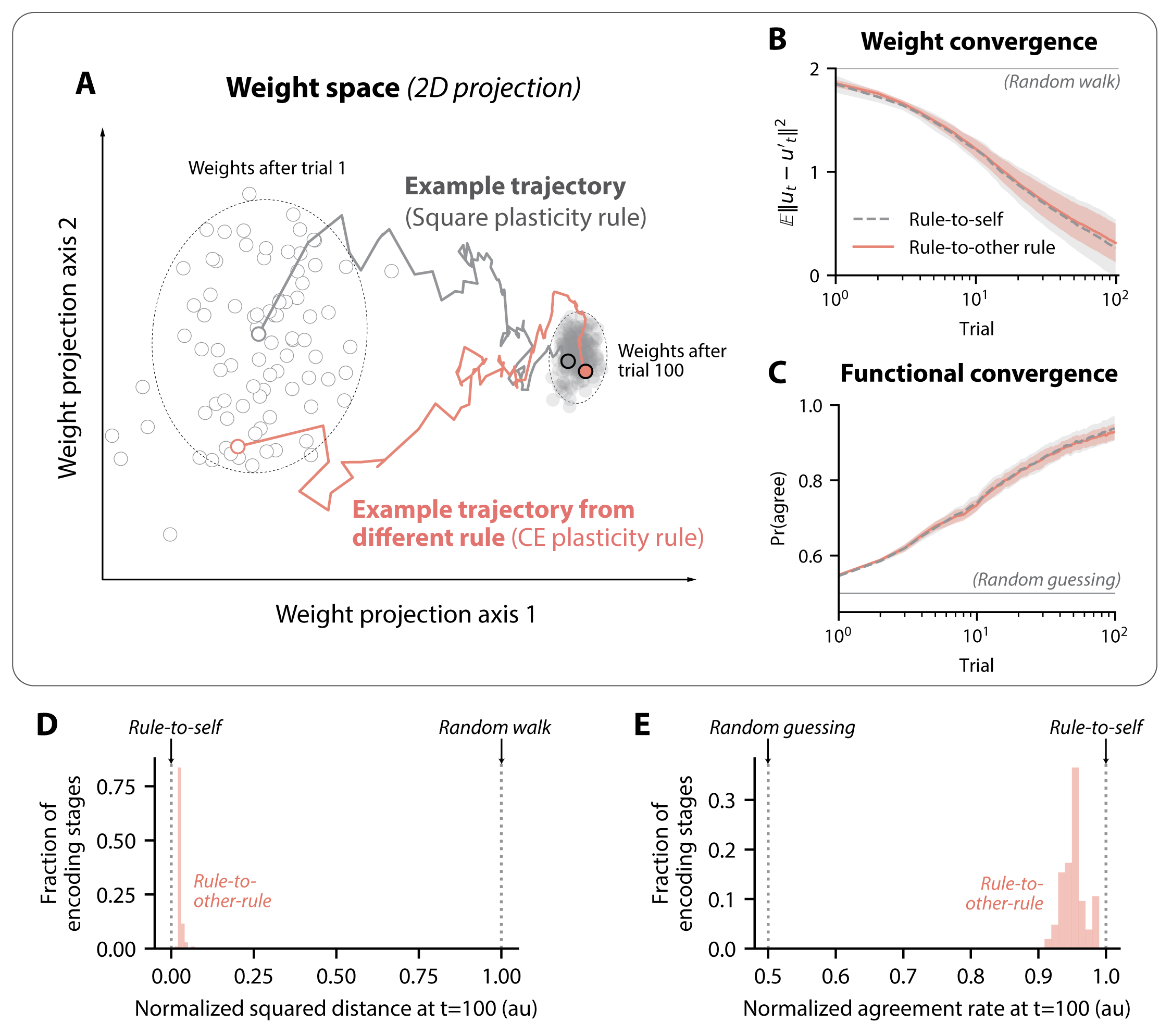

Supplement: S3 Fig — A. Example learning trajectories in weight space. For an example plasticity rule (square) operating on an example representational space (ResNet50/avgpool), weight vectors near the beginning (open dots) and end (closed dots) of different learning simulations for a subtask are shown. One example trajectory (gray) for a single simulation is shown. In red is a trajectory from a different rule. B. Weight convergence. For the example encoding stage in panel A, the expected (over subtasks, simulations) squared pairwise ℓ2 distance in weight space between independent simulations from all n = 7 plasticity rules (red) remains close to the lower limit (gray), across trials. Shaded regions are the ± standard deviation over rules. C. Functional convergence. The probability that two distinct rules (red) generate the same behavioral prediction for a random, held-out test image is shown, and compared to the probability a rule agrees with itself across two independent simulations (dashed gray). D. Summary of weight convergence across all encoding stages. Shown is a normalized rule-to-other rule distance metric (normalized between random walk; rule-to-self) at trial 100 for all encoding stages (median: 0.02, max: 0.07). D. Summary of functional convergence. A normalized functional convergence metric (norm. between random guessing, rule-to-self) for all encoding stages is shown (median: 0.95, min: 0.92). See Section 2.3 in S1 Appendix for details. (TIF) [file pcbi.1011713.s003.tif]

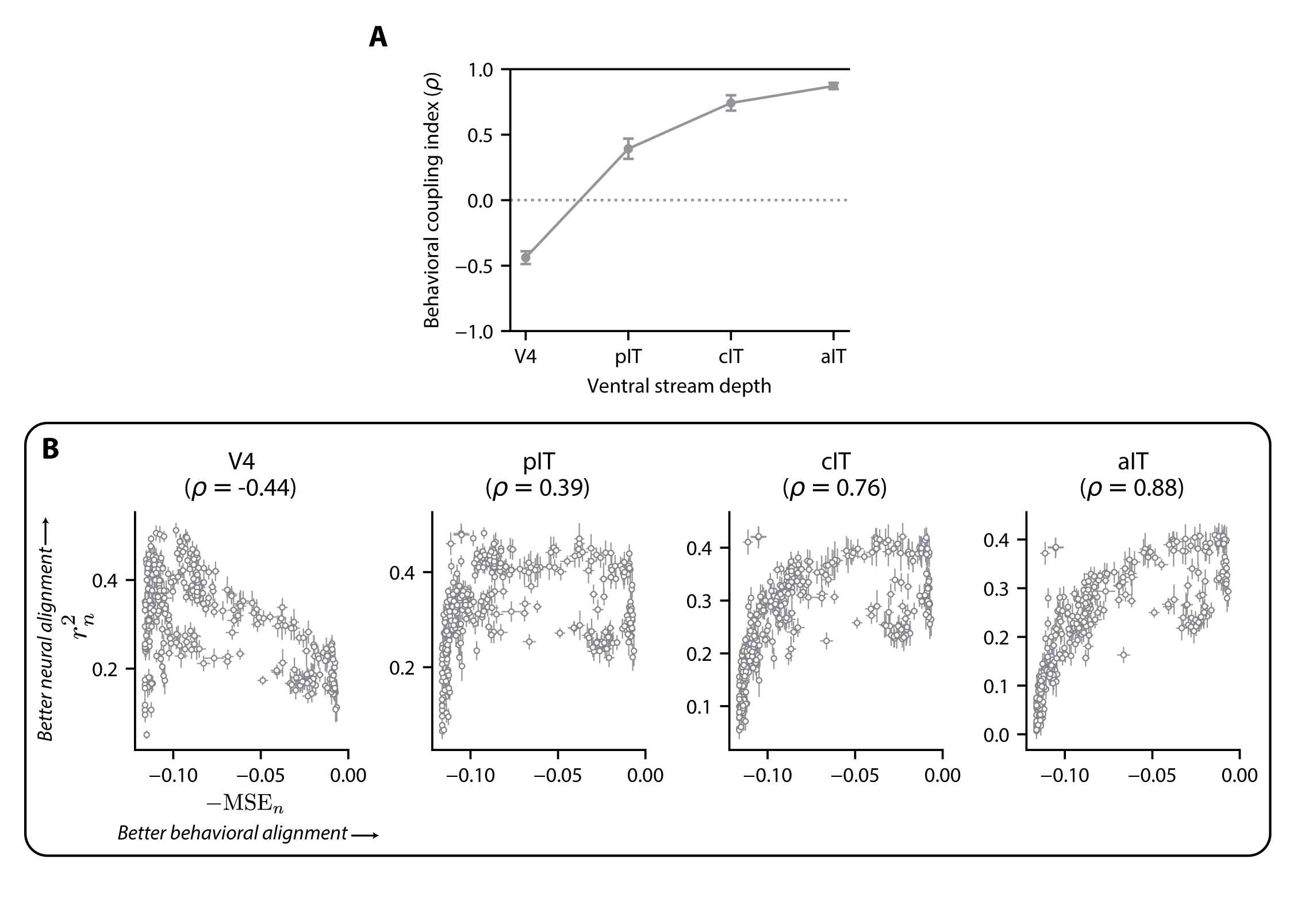

Supplement: S4 Fig — A. Human learning similarity is increasingly predicted by alignment with deeper subregions of the ventral stream. The behavioral coupling index (ρ) is the rank correlation coefficient between neural alignment (rn2) and behavioral alignment (−MSEn, for Experiment 1). Errorbars are the approximate 95% confidence interval over random train/test image split-halves, for the neural alignment metric. B. Subregion-wise neural alignment vs. behavioral alignment. Each point corresponds to a specific encoding stage (n = 344 total). The y-axis is a previously described neural alignment metric (rn2); a higher value means the encoding stage is a better linear predictor of image-driven firing rates in the corresponding brain region. The x-axis reflects an encoding stage’s expected (over plasticity rules) behavioral alignment with humans, as evaluated by Experiment 1; higher means more similar to humans. The behavioral coupling index ρ is the Spearman correlation coefficient between the two metrics, over these encoding stages. Errorbars are the standard deviation over train/test image splits (y-axis) and plasticity rules (x-axis). See Section 3 in S1 Appendix for details. (TIF) [file pcbi.1011713.s004.tif]

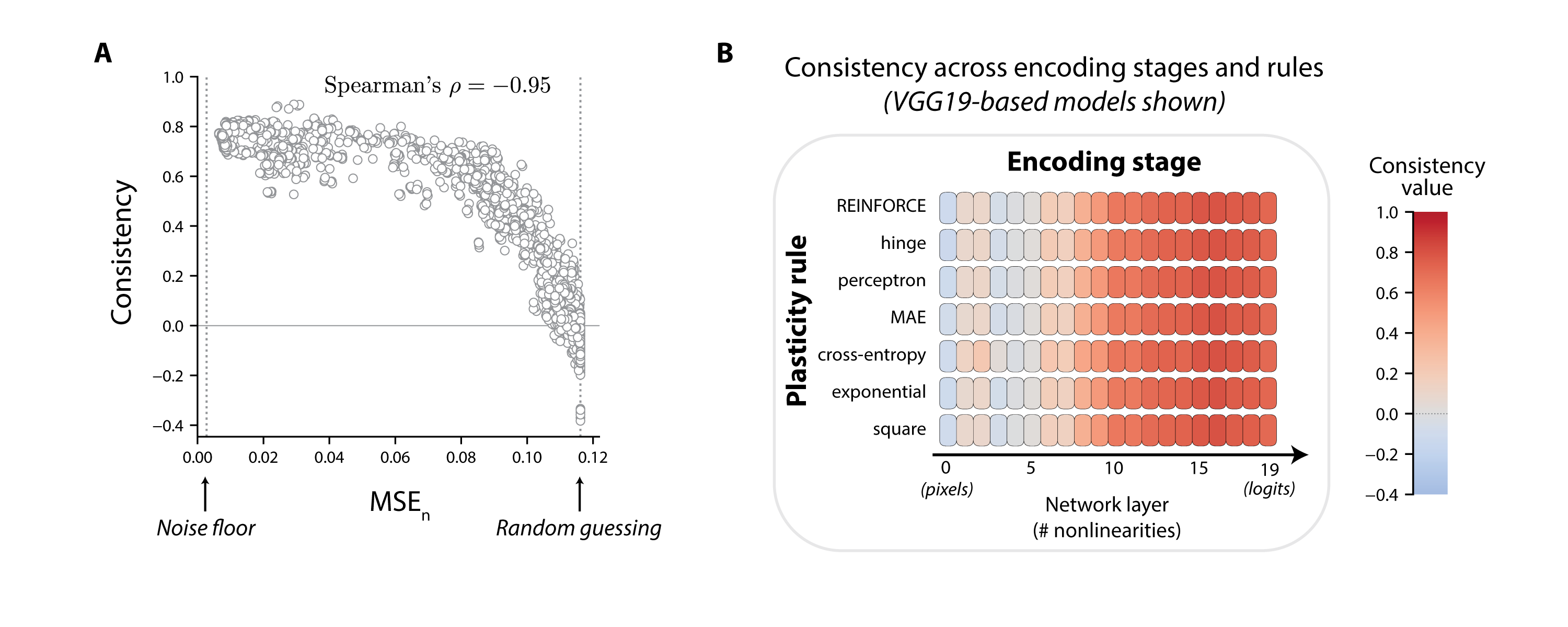

Supplement: S5 Fig — A. Correspondence of consistency- and MSEn-based model rankings. As described in the main text, the consistency score for a model is the rank-order correlation between its trial-averaged subtask accuracies and that of humans. Over the models tested in this study (dots), their MSEn scores (x-axis) had a semilinear relationship with their consistency scores (y-axis) on the primary benchmark. Overall, we found the models’ rankings based on MSEn to be highly similar to their rankings based on consistency (Spearman’s rank correlation coefficient ρ = −0.95); note lower MSEn means more aligned with humans. B. Relative contributions of encoding stage and plasticity rule to consistency. Shown are consistency scores for a representative collection of models (all learning models based on VGG19 encoding stages). Similar to our findings for MSEn, we found that the choice of plasticity rule had a negligible effect on a model’s consistency (0.25% of explained variance in two-way ANOVA; see Section 2.1 in S1 Appendix), while encoding stages had a dominant effect (>99% explained variance). (TIF) [file pcbi.1011713.s005.tif]
