# Supplementary material for: How well do rudimentary plasticity rules predict adult visual object learning?
Source: PLoS Comput Biol. 2023 Dec 11;19(12):e1011713. doi: 10.1371/journal.pcbi.1011713 (PMC10754461; doi:10.1371/journal.pcbi.1011713)
Supplement: S1 Table — Each plasticity rule in this study guides a weight update w→c←w→c+δ→c, based on the current input x→∈Rd, the selected choice c ∈ {1, …, C}, and the subsequent environmental reward r ∈ [−1, 1]. Here, we provide the equation for the update term δ→c for each plasticity rule. (PDF) [file pcbi.1011713.s006.pdf]

S1 Table. Summary of plasticity rules.

Each plasticity rule can be understood by the update $\vec{\delta}_i$ it generates for each weight vector $\vec{w}_i \leftarrow \vec{w}_i + \vec{\delta}_i$, based on the current input $\vec{x} \in \mathbb{R}^d$, the selected choice $c \in \{0, 1, ...C\}$, and the subsequent environmental reward $r \in [-1, 1]$. Each plasticity rule is parameterized by a learning rate α, between 0 and 1. These equations assume that the input $\vec{x}$ has a bounded norm of $\|\vec{x}\| \leq 1$.

**Plasticity rule**	**Weight update for selected choice** ($\vec{\delta}_c$)
Perceptron	$\vec{\delta}_c = \begin{cases} \alpha r \vec{x} & \text{if } r\left(\vec{w}_c \cdot \vec{x}\right) \leq 0 \\ 0 & \text{else} \end{cases}$
Hinge	$\vec{\delta}_c = \begin{cases} \alpha r \vec{x} & \text{if } r\left(\vec{w}_c \cdot \vec{x}\right) < 1 \\ 0 & \text{else} \end{cases}$
MAE	$\vec{\delta}_c = \begin{cases} \alpha \vec{x} & \text{if } \left(\vec{w}_c \cdot \vec{x}\right) < r \\ -\alpha \vec{x} & \text{else} \end{cases}$
Square	$\vec{\delta}_c = \alpha \left(r - \vec{w}_c \cdot \vec{x}\right) \vec{x}$
Exponential	$\vec{\delta}_c = \alpha r \exp\left(-r \vec{w}_c \cdot \vec{x}\right) \vec{x}$
Cross-entropy	$\vec{\delta}_c = \alpha r \left(\frac{4}{1+\exp\left(r \vec{w}_c \cdot \vec{x}\right)}\right) \vec{x}$
REINFORCE	$\vec{\delta}_c = \alpha r \left(1 - \exp\left(\vec{w}_c \cdot \vec{x} - \log\left(\sum_{j=1}^{C} \exp\left(\vec{w}_j \cdot \vec{x}\right)\right)\right)\right) \vec{x}$ $\vec{\delta}_{i \neq c} = -\alpha r \exp\left(\vec{w}_i \cdot \vec{x} - \log\left(\sum_{j=1}^{C} \exp\left(\vec{w}_j \cdot \vec{x}\right)\right)\right) \vec{x}$

Except for REINFORCE, each rule above only changes the weight vector for the selected choice c; the other weight vector(s) are left unchanged ($\vec{\delta}_{i \neq c} = 0$). Additionally, the Exponential plasticity rule performs a weight normalization step (not shown): if the norm of the weights exceed a certain threshold ($\|\vec{w}_i\| > B$) , the weights are projected to the closest weight vector $\vec{w}'_i = \mathbf{argmin}_{u;\|u\|=B} \left(\|\vec{u} - \vec{w}_i\|\right)$ with $\|\vec{w}'_i\| = B$. In this study, we chose $B = 10$.
