# Supplementary material for: How well do rudimentary plasticity rules predict adult visual object learning?
Source: PLoS Comput Biol. 2023 Dec 11;19(12):e1011713. doi: 10.1371/journal.pcbi.1011713 (PMC10754461; doi:10.1371/journal.pcbi.1011713)
Supplement: S1 Appendix — (PDF) [file pcbi.1011713.s007.pdf]

S1 Appendix: Supplementary Methods

Contents

1 Additional behavioral metrics **1**
- 1.1 Subtask consistency . . . 1
- 1.2 Individual variability in overall learning ability . . . 2
  - 1.2.1 Permutation test for individual variability in overall learning ability . . . 2
  - 1.2.2 Testing whether specific humans outperform a model . . . 2

2 Analysis of differences between learning models **3**
- 2.1 Effect of model choices on human behavioral similarity . . . 3
- 2.2 Differences between plasticity rules in image-averaged behavior . . . 3
- 2.3 Differences between plasticity rules in weights and image-by-image behavior . . . 4
  - 2.3.1 Weight convergence analysis . . . 4
  - 2.3.2 Functional convergence analysis . . . 5

3 Comparison of neural and behavioral alignment in baseline learning models **5**
- 3.1 Noise-corrected neural alignment metric . . . 5
- 3.2 Behavioral coupling index . . . 6

1 Additional behavioral metrics

1.1 Subtask consistency

In our primary benchmark, we measured human learning over 64 distinct subtasks, each consisting of 100 trials. For each subtask, the trial-averaged accuracy is a measure of the overall "difficulty" of learning that subtask, ranging from chance (0.5; no learning occurred over 100 trials) to perfect one-shot learning (0.995, perfect performance after a single example). For each of the 64 subtasks, one may estimate their trial-averaged performances (obtaining a length 64 "difficulty vector"), and use this as the basis of comparison between two learning systems (e.g. humans and a specific model).

To do so, we computed Spearman's rank correlation coefficient (ρ) between a model's difficulty vector and the human's difficulty vector. The value of ρ may range between -1 and 1. If $\rho = 1$, the model has the same ranking of difficulty between the different subtasks (i.e., finds the same subtasks easy and hard). If $\rho = 0$, there is no correlation in the rankings.

In addition to computing ρ between each model and humans, we estimated the ρ that would be expected between two independent repetitions of the experiment we conducted here (i.e., an estimate of experimental reliability in measuring this difficulty vector). To do this, we took two independent bootstrap resamples of the experimental data, calculated their respective difficulty vectors, and computed the ρ between them. We repeated this process for $B = 1,000$ bootstrap iterations, and thereby obtained the expected distribution of experimental-repeat ρ.

1.2 Individual variability in overall learning ability

In this work, we focused primarily on *subject-averaged* measurements of human learning. However, individual subjects may also systematically differ from each other. We aimed to investigate whether any such differences existed in learning behavior for the subtasks we tested in this study.

Here, we attempted to reject the null hypothesis that all subjects had the same learning behavior. To do so, we tested whether there were statistically significant differences in *overall learning performance* between individuals – that is, whether some individuals were "better" or "worse" learners. If this was the case, this implies individuals differ (at least in terms of overall learning performance), and the null hypothesis could be rejected.

1.2.1 Permutation test for individual variability in overall learning ability

To test this null hypothesis, we identified a subset of human subjects who conducted all 64 subtasks in the primary, high-variation benchmark (n=22 subjects). For each subject, we computed their "overall learning performance", which was their empirically observed average performance over all n=64 subtasks. That is, for subject s, we computed:

$$\hat{G}_s = \frac{1}{64}\sum_{\text{i=1}}^{64} \hat{g}_{is}$$

Where $\hat{g}_{is}$ is the trial-averaged performance on subtask i, for subject s. The value of $\hat{G}_s$ is a gross measure of the subject's ability to learn the objects in this study, ranging from 0.5 (no learning on all subtasks) to 0.995 (perfect one-shot learning on all subtasks). In total, we computed n=22 estimates of $\hat{G}_s$ (one for each subject in this analysis).

We then computed the sample variance over the various $\hat{G}_s$:

$$\hat{\sigma}^2 = \frac{1}{S-1}\sum_{s=1}^{S}(\hat{G}_s - \bar{G})^2$$

Where $\bar{G}$ is the mean of overall lifetime performances. Intuitively, $\hat{\sigma}^2$ is high if individuals differ in their overall learning performance, and is low if all individuals have the same overall learning performance (as would be the case under the null hypothesis).

We performed a permutation test on $\hat{\sigma}^2$ to test whether it was significantly higher than would be expected under the null hypothesis, permuting the assignments of each $\hat{g}_{is}$ to each subject s. For each permutation, we computed the replication test statistic $\tilde{\sigma}^2$ (using the same formulas above, on the permuted data). We performed $P = 10,000$ permutation replications, then computed the one-sided achieved significance level by counting the number of replication test statistics greater than the actual, experimentally observed value $\hat{\sigma}^2$.

1.2.2 Testing whether specific humans outperform a model

To test whether a specific human has significantly higher overall learning abilities than a specific model (over the subtasks tested in this study), we performed Welch's t-test for unequal variances on the overall learning performance, $\hat{G}$ (defined above). That is, for a specific subject s and model m, we attempted to reject the null hypothesis that $\hat{G}_s \leq \hat{G}_m$.

We adjusted for multiple comparisons using the Bonferroni correction (using the total number of pairwise comparisons we made between a model m and specific subjects s).

2 Analysis of differences between learning models

2.1 Effect of model choices on human behavioral similarity

Each model in this study was defined by two components (the encoding stage and the plasticity rule). We wished to evaluate the effect of each of these components in driving the similarity of the model to human behavior. For example, it was possible that all models with the same encoding stage had the same learning score, regardless of which plasticity rule they used (or vice versa).

To test for these possibilities, we performed a two-way ANOVA over all observed model scores (in MSE_n) computed in this study, using the encoding stage and plasticity rule as the two factors, and MSE_n as the dependent variable. By doing so, we were able to estimate the amount of variation in model scores that could be explained by each individual component, and thereby gauge their relative importance. We briefly describe the procedure for this analysis below. First, we wrote the MSE_n score of each model as a combination of four variables:

$$\text{MSE}_n(\text{encoding stage } i, \text{rule } j) = \mu + e_i + r_j + \gamma_{ij}$$

Where μ is the average MSE_n score, over all models. The variables e_i and r_j encode the value of the average difference from μ given encoding stage i and rule j, respectively. Any remaining residual is assigned to γ_{ij} (i.e. corresponding to any interaction between rule and encoding stage). The importance of each model component could be assessed by calculating the proportion of variation in model scores that could be explained by the selection of component alone.

2.2 Differences between plasticity rules in image-averaged behavior

Our primary learning metric (MSE_n) compares the subtask × trial behavioral matrix produced by a model ($\hat{M}$) and humans ($\hat{H}$) (see Materials and Methods). We found that learning models based on the same encoding stage – i.e. learning models that differ only in their plasticity rule – tended to have highly similar MSE_n scores (see Fig 5).

This does not necessarily imply that these learning models generate the same behavioral predictions: it is possible for two learning models to have the same MSE_n score with respect to humans, but distinct behaviors. Geometrically, this can be understood by the fact that $\text{MSE}_n(\hat{M}, \hat{H})$ is directly proportional to the squared ℓ_2 distance between a model and humans in the vector space of behavioral measurements, (where each axis corresponds to an accuracy measurement taken at a specific trial and subtask; see Figure 6A). Two models which are "equidistant" from humans in this space (i.e., have the same MSE_n score) may or may not be in the same location.

For each encoding stage, we therefore directly quantified differences between different plasticity rules (n=7) by calculating the average squared pairwise distance between each of their corresponding behavioral matrices. To estimate these squared pairwise distances, we used a minor variation of the previously described MSE_n metric that performs bias-correction on both arguments (note that $\text{MSE}_n(\hat{M}, \hat{H})$ performs bias correction only with respect to the model $\hat{M}$). The resultant metric (which we refer to as MSE_u) is symmetric in both arguments, and is an unbiased estimator of the expected squared differences between two models, given zero sampling noise:

$$\text{MSE}_u(\hat{M}, \hat{M}') = \frac{1}{S \cdot T} \sum_{s=1}^{S} \sum_{t=1}^{T} (\hat{M}_{st} - \hat{M}'_{st})^2 - \Big(\hat{\sigma}^2(\hat{M}_{st}) + \hat{\sigma}^2(\hat{M}'_{st})\Big) \tag{1}$$

$$\mathbf{E}[\text{MSE}_u(\hat{M}, \hat{M}')] = \frac{1}{S \cdot T} \|M - M'\|_2^2 \tag{2}$$

Where $M = \mathbf{E}[\hat{M}]$ is the true, expected behavior of the model, $\hat{\sigma}^2(\hat{M}_{st})$ is the previously described estimator of the measurement variance of $\hat{M}_{st}$ (Equation 4, in the Materials and Methods), and $\|\cdot\|_2^2$ is the Frobenius matrix norm.

The average distance of each rule to humans (also measured by MSE_u) provides a natural reference point for assessing the extent to which these models are "similar" (or not) to each other. We calculated, for each encoding stage, the average rule-to-other-rule MSE_u as a percentage of its average rule-to-human MSE_u. We estimated the median percentage (across n=344 encoding stages) was 0.04%; the maximum was 8.26%.

2.3 Differences between plasticity rules in weights and image-by-image behavior

In general, each plasticity rule in this study drives non-identical updates to the weights of the tunable decision stage. However, we observed that different plasticity rules tend to achieve similar benchmark scores with respect to humans (see Fig 5) and generate similar image-averaged behavioral predictions across trials (see Fig 6). One natural next question to ask is: do different plasticity rules differ at finer levels of analysis?

We sought to answer this question in two ways: first, by measuring whether the weights updated by two different rules tend to be close in *weight space* at the end of learning ("weight convergence"), and second, by measuring whether different rules generate similar *image-by-image* behavioral predictions ("functional convergence").

First, we briefly re-describe the learning procedure for any given model. We then describe each analysis in the sections that follow.

Each model has a collection of d-dimensional weight vectors $\vec{w}_1, ..., \vec{w}_C$ associated with the C possible choices the model may make. Given a d-dimensional image representation $\vec{x}$, each weight vector computes a choice preference by taking dot products with the current image representation ($\vec{x} \cdot \vec{w}_i$). Finally, a behavioral choice is generated by selecting the largest possible choice preference (breaking ties randomly).

In this study, because the number of choices is $C = 2$, the state of the model may be fully summarized by a single weight vector, which we now denote by $\vec{w} = \vec{w}_1 - \vec{w}_2$. This weight vector can be understood as defining a linear decision boundary (hyperplane) in the d-dimensional representational space of images. This weight vector $\vec{w}$ (i.e. the learning state of the model) is updated over trials, and different plasticity rules in general drive different updates.

2.3.1 Weight convergence analysis

In this analysis, the goal was to compare weight vectors after being driven by sequences of updates guided by different plasticity rules. Because the norm of the weights $\|\vec{w}\|$ does not affect the behavior of the model (in that positively scaling $\vec{w}$ does not change the orientation of the associated hyperplane), we chose to compare *normalized* weight vectors, which we denote by $\vec{u} = \frac{\vec{w}}{\|\vec{w}\|}$. These normalized vectors encode the orientation of decision hyperplanes in the feature space of the model.

To compare two normalized weight vectors $\vec{u}$ and $\vec{u}'$, we used the (squared) ℓ_2 distance $\|\vec{u} - \vec{u}'\|^2$. This value is zero if $\vec{u}$ and $\vec{u}'$ are identical, is 2 if they are orthogonal, and is at most 4 (if opposite in sign but otherwise equal). For each encoding stage, we estimated the expected value of $\|\vec{u} - \vec{u}'\|^2$ at the end of learning, where $\vec{u}$ and $\vec{u}'$ are the (normalized) weight vectors generated by different plasticity rules after trial 100. The expectation was taken over all possible pairs of plasticity rules (n=$\binom{7}{2}$ pairs), over all subtasks of Experiment 1 (n=64 subtasks), and over randomly sampled trial sequences for each subtask (here, n=4 random trial sequences were used). We generated $\vec{u}$ and $\vec{u}'$ samples from separate, independent trial sequences (an even finer-grained analysis might compare them on identical trial sequences).

We refer to the average statistic above as the "expected rule-to-other-rule distance". Intuitively, if this statistic is low, then all rules tend to produce decision hyperplanes that are oriented similarly by the end of learning, given any subtask. Note that this statistic reflects the average similarity over all possible pairs of rules; we did not examine how specific pairs of rules might differ.

We normalized the (expected) rule-to-other-rule distance against two natural reference points: 1) the expected rule-to-self distance, where two weight vectors generated by the same rule are compared to each other (which provides a natural floor), and 2) the expected rule-to-random-walk distance, where the distance to weights from a random walk is taken (which provides a natural ceiling). We show how all three statistics vary across trials in Panel B in S3 Fig, for an example encoding stage.

Finally, we use those statistics to report a single, normalized rule-to-other-rule distance between 0 and 1 (using the reference points above). We computed this normalized statistic for all encoding stages, and show them in a histogram in Panel D in S3 Fig. Across encoding stages, the median value was 0.02; the maximum was 0.07.

2.3.2 Functional convergence analysis

Beyond comparing different plasticity rules on the basis of their weights, we also compared them in terms of their image-by-image behavioral predictions – a level of analysis that is finer grained than the *image-averaged* behavioral statistics in our benchmarks. The analysis procedure was nearly identical to the one described above. Here, we simply replaced the use of the ℓ_2 distance between weights with the *probability of behavioral agreement* between two plasticity rules, as evaluated on a distribution of held out test images.

The distribution of test images varied by subtask; we kept n=100 randomly selected images (50 from each object category) fully held out from the training sequences. Following the end of the training sequence (i.e. after 100 trials), we calculated the fraction of images where the models associated with the two rules agreed (regardless of whether they were correct or incorrect).

As before, we normalized this measure against two reference points: first, the probability of agreement expected by an unbiased random guesser (always 0.5, providing a floor), and the probability of a rule agreeing with itself (between two independently sampled trial sequences of a subtask), which provides a ceiling.

For each encoding stage, we report the expected probability of agreement (over all pairwise rule comparisons) on a normalized scale between 0.5 (random guessing) and 1 (the self-agreement probability). We show the results in Panel E of S3 Fig. The median normalized agreement rate (over encoding stages) was 0.95; the minimum was 0.92.

3 Comparison of neural and behavioral alignment in baseline learning models

3.1 Noise-corrected neural alignment metric

An image-computable encoding stage (which maps pixel images to vectors $\vec{x} \in \mathbb{R}^d$) may be interpreted as a candidate hypothesis for the relationship between an incoming retinal image and the evoked pattern of neural firing rates in a particular brain region.

Using a previously published electrophysiological dataset which measured firing rates in the ventral stream in response to naturalistic images in n=2 male macaques [1], we estimated the extent to which all encoding stages in this study (n=344) aligned with the neural representations in four distinct subregions of increasing processing depth. The images in this study are comparable to the ones in the present study, in that they involve 3D objects rendered on natural backgrounds with random viewing parameters, all in grayscale.

We used a previously described neural alignment metric for this purpose [3]. We give an overview of the procedure here: given an image-computable encoding stage (which re-represents images as d-dimensional vectors), one computes the encoding stage's responses to the $N = 2,560$ stimulus images in the experiment (these images are from the `private` split of the `HvM` dataset, from the Brain-Score platform [2]). The resultant matrix ($\mathbb{R}^{N,d}$) is compared to a matrix of standardized neural rate responses ($\mathbb{R}^{N,p}$), where p is the number of recorded (multi-)unit sites in the brain region being considered (e.g. V4). To perform this comparison, a linear regression model is first built using partial least squares (PLS) regression (with

n=25 PLS components), using a random subsample of the data consisting of 90% of the images. Then, the remaining, unused 10% of the images are used to evaluate the quality of the regression model: squared Pearson correlation coefficients r^2 are estimated between the responses of each recorded neural unit and the regression model of that neural unit.

In principle, each r^2 value ranges between 0 and 1, but because there is measurement uncertainty in the estimates of the firing rate of each neuron to each image ("noise"), there is an upper bound to the r^2 value that may be expected by even a "perfect" model of that neuron (the "noise ceiling"). We therefore normalized each r^2 value by estimates of the noise ceiling for each neuron. These noise ceiling estimates were based on the following identity for the squared population correlation coefficient (denoted by ρ^2). Given the random variable I and two random variables X, Y which are conditionally independent given I:

$$\rho^2_{X,Y} = \frac{\mathbf{cov}^2(X,Y)}{\mathbf{var}(X)\mathbf{var}(Y)} \tag{3}$$

$$= \frac{\mathbf{cov}^2(\mathbf{E}[X|I), \mathbf{E}[Y|I])]}{(\mathbf{E}[\mathbf{var}(X)|I] + \mathbf{var}(\mathbf{E}[X|I]))\,\mathbf{var}(Y)} \tag{4}$$

$$\leq \frac{\mathbf{var}(\mathbf{E}[X|I])\mathbf{var}(\mathbf{E}[Y|I])}{(0 + \mathbf{var}(\mathbf{E}[X|I]))\mathbf{var}(Y)} \tag{5}$$

$$\leq \frac{\mathbf{var}(\mathbf{E}[Y|I])}{\mathbf{var}(Y)} \tag{6}$$

The first line is by definition. The second line applies the law of total (co)variance and uses the conditional independence of X and Y. The third line uses the Cauchy-Schwarz inequality (for covariance). In the present case, X denotes a model, Y denotes an experimental estimate of the rep-averaged firing rate for a neuron, and I denotes an image. For each neuron, we plugged in unbiased estimates for each term in the equation above to estimate its noise ceiling.

For each neuron, we repeated this process over n=10 random train/test image splits (estimating noise ceilings on each test split). We refer to the average normalized r^2 values (averaged over splits, over neural units) as r^2_n. In S4 Fig, we report r^2_n values for four nonoverlapping subsets of neural units recorded in [1], each from different subregions (V4, pIT, cIT, and aIT). In the procedure outlined above, we built two PLS regression models for each encoding stage: one based solely on units from V4 (n=88 units) and one based on units pooled from all three IT subregions (n=168 total; n=76 pIT, n=75 cIT, n=17 aIT). We calculated r^2_n values for the three IT subregions separately.

3.2 Behavioral coupling index

We sought to compare an encoding stage's alignment with various neural subregions (as quantified by r^2_n) with its ability to yield learning behaviors similar to that of humans. An encoding stage itself does not generate testable learning predictions; it must be combined with a plasticity rule. For each encoding stage, we took its average (over the n=7 tested plasticity rules) MSE_n score on our primary benchmark (from Experiment 1). Then, for each subregion, we computed the Spearman rank-correlation coefficient ρ between the neural alignment metric r^2_n and the (negative) average MSE_n score over all n=344 encoding stages (see Panel B in S4 Fig). We refer to this correlation ρ as the "behavioral coupling index". A positive value of ρ for a subregion means that an encoding stage's neural predictivity for that subregion is *positively correlated* with its (rule-averaged) human behavioral predictivity, and a negative value implies the two are negatively correlated. Note that we used the negative MSE_n simply for presentation purposes, so a positive ρ indicates a positive relationship, as desired. We found that the coupling index increased across successive stages of the ventral stream (see Panel A in S4 Fig).

References

[1] Majaj NJ, Hong H, Solomon EA, DiCarlo JJ. Simple Learned Weighted Sums of Inferior Temporal Neuronal Firing Rates Accurately Predict Human Core Object Recognition Performance. Journal of Neuroscience. 2015;35(39):13402–13418. doi:10.1523/JNEUROSCI.5181-14.2015.

[2] Schrimpf M, Kubilius J, Lee MJ, Ratan Murty NA, Ajemian R, DiCarlo JJ. Integrative Benchmarking to Advance Neurally Mechanistic Models of Human Intelligence. Neuron. 2020;108(3):413–423. doi:10.1016/J.NEURON.2020.07.040.

[3] Yamins DLK, Hong H, Cadieu CF, Solomon EA, Seibert D, DiCarlo JJ. Performance-optimized hierarchical models predict neural responses in higher visual cortex. Proceedings of the National Academy of Sciences of the United States of America. 2014;111(23):8619–24. doi:10.1073/pnas.1403112111.
